# Supplementary material for: Anakinra or tocilizumab in patients admitted to hospital with severe covid-19 at high risk of deterioration (IMMCoVA): A randomized, controlled, open-label trial
Source: PLoS One. 2023 Dec 29;18(12):e0295838. doi: 10.1371/journal.pone.0295838 (PMC10756513; doi:10.1371/journal.pone.0295838)
Supplement: S1 Table — (DOCX) [file pone.0295838.s001.docx]

**S1 Table. Inclusion and exclusion criteria**

1) Age ≥18 years

2) Laboratory-confirmed SARS-CoV-2 infection as determined by polymerase chain reaction (PCR) or other commercial or public health assay < 3 days prior to screening

3) SARS-CoV-2 infection with duration at least 7 days (as determined by onset of symptoms)

4) 5 liters/minute of Oxygen for at least 8 hours to maintain SpO2 at ≥93%. A shorter duration is also accepted if presentation is acute, and the patient needs more than 10 l liters/minute of Oxygen to maintain SpO2 at ≥93%.

5) CRP > 70 mg/L with no non-SARS-Cov2 infections. Values measured up to 48 hours before inclusion are accepted.

6) Ferritin > 500 µg/L. Values measured up to 48 hours before inclusion are accepted.

7) At least two points on a scale of 0-3 where 1 point is awarded for each value of; lymphocytes < 1x 10(9)/L; D-dimer ≥ 0.5 mg/L and; Lactate Dehydrogenase ≥ 8 microkatal/L. The values do not have to be concurrently positive and may be up to 3 days old at inclusion.

8) Ability to provide informed consent signed by study patient

9) Willingness and ability to comply with study-related procedures/assessments

10) In fertile females, willing to comply with effective contraceptive methods for up to 3 months after last dose of study drug. These may include birth control pills, surgical sterilization of patient or partner, intrauterine device or condoms, but not birth control pills which may increase risk of deep venous thrombosis during COVID infections. Non-fertile woman is defined as more than 12

Exclusion criteria

1. Pregnancy or breast feeding.
2. Ongoing or completed mechanical ventilation.
3. In the opinion of the investigator, unlikely to survive for >48 hours from screening.
4. In the opinion of the investigator, expected overall survival due to other comorbidities less than 3 months.
5. Severe renal dysfunction eGFR < 30 ml/min.
6. Medical history including chronic liver disease with inflammation, fibrosis or cirrhosis including underlying diseases such as alcoholic liver disease, non-alcoholic fatty liver disease, chronic viral hepatitis, alcoholic liver disease, autoimmune liver disease, hemochromatosis, Wilson’s disease, alpha-1 antitrypsin deficiency, cholangitis, or carcinoma.
7. Uncontrolled hypertension Systolic BP >180 mm Hg, Diastolic BP > 110 mm Hg
8. History of hypersensitivity to the study drugs
9. Presence of any of the following abnormal laboratory values at screening: absolute neutrophil count (ANC) less than 2 x 10^9^/L, aspartate aminotransferase (AST) or alanine aminotransferase (ALT) greater than 5 x upper limit of normal (ULN), platelets <100 x 10^9^/L
10. Treatment with anakinra, anti-IL 6, anti-IL-6R antagonists, Janus kinase inhibitors (JAKi) in the past 30 days or plans to receive during the study period
11. Current treatment with conventional synthetic disease-modifying antirheumatic drugs (DMARDs)/immunosuppressive agents
12. Use of chronic oral corticosteroids for a non-COVID-19-related condition in a dose higher than prednisone 10 mg or equivalent per day. Ongoing acute treatment for COVID-19 with any peroral or iv steroid is permitted for up to five days before inclusion. Chronic or acute treatment with inhaled steroids is also permitted.
13. History of, or current autoimmune or inflammatory systemic or localized disease(s) other than rheumatoid arthritis
14. Acute systemic infection; verified by blood cultures systemic bacterial infection, systemic fungi-infection or prosthesis-related infection
15. History of stem-cell or solid organ transplantation
16. Known active tuberculosis (TB), history of incompletely treated TB, suspected or known extrapulmonary TB, suspected or known systemic bacterial or fungal infections
17. Diagnosis of, or suspicion of HIV infection, acute hepatitis A and/or chronic hepatitis B and/or C
18. Previous history of gastrointestinal ulceration or diverticulitis.
19. Patients who have received immunosuppressive antibody therapy within the past 3 months, including intravenous immunoglobulin or plans to receive during the study period
20. Participation in any clinical research study evaluating an investigational product (IP) or therapy within 3 months and less than 5 half-lives of IP prior to the screening visit. The use of remdesivir is permitted.
21. Any physical examination findings and/or history of any illness that, in the opinion of the study investigator, might confound the results of the study or pose an additional risk to the patient by their participation in the study

Experimental treatments other than those specified in guidelines or off-label use of marketed medications for covid-19 were prohibited within 3 months and less than 5 half-lives of investigational product prior to screening.
