# Supplementary material for: Anakinra or tocilizumab in patients admitted to hospital with severe covid-19 at high risk of deterioration (IMMCoVA): A randomized, controlled, open-label trial
Source: PLoS One. 2023 Dec 29;18(12):e0295838. doi: 10.1371/journal.pone.0295838 (PMC10756513; doi:10.1371/journal.pone.0295838)
Supplement: S2 Table — (DOCX) [file pone.0295838.s002.docx]

**S2 Table. Severe adverse events by day 60**

| SAE | UC (n=27) | Anakinra (n=28) | Tocilizumab (n=22) |
| --- | --- | --- | --- |
|  |  |  |  |
| Any SAE | 32 | 24 | 15 |
| Any SAE, no of patients | 13 | 13 | 6 |
| Septicaemia | 1 (3.1) | 2 (8.3) | 0 |
| Coronary artery dissection | 1 (3.1) | 0 | 0 |
| GI bleeding | 1 (3.1) | 0 | 0 |
| Pancreatitis | 1(3.1) | 0 | 0 |
| Bowel perforation | 0 | 1 (0.04) | 0 |
| Bacterial pneumonia | 2 (6.3) | 0 | 0 |
| Post-Covid syndrome | 0 | 1 (0.04) | 0 |
| Other infection | 1 (3.1) | 0 | 1 (6.7) |
| Positive test for infectious agents | 0 | 0 | 2 (13.3) |
| Death | 3 (9.4) | 3 (12.5) | 2 (13.3) |
| Delirium | 1 (3.1) | 0 | 0 |
| Critical illness polyneuropathy | 1 (3.1) | 0 | 0 |
| Other severe neurological manifestation | 1 (3.1) | 0 | 0 |
| Acute Kidney failure | 1 (3.1) | 1 (0.04) | 0 |
| Pulmonary embolus | 3 (9.4) | 1 (0.04) | 0 |
| Lung fibrosis | 0 | 0 | 1 (6.7) |
| Aspiration pneumonitis | 1 (3.1) | 0 | 0 |
| Pulmonary hypertension | 1 (3.1) | 0 | 1 (6.7) |
| Pneumothorax | 1 (3.1) | 1 (0.04) | 0 |
| Pneumomediastinum | 0 | 0 | 1 (6.7) |
| Circulatory failure | 1 (3.1) | 0 | 0 |
| Deep Venous Thrombosis | 0 | 1 (0.04) | 0 |
| Respiratory failure | 10 (31.3) | 13 (52.0) | 7 (46.7) |
| Other* | 1 (3.1) | 0 | 0 |

Serious adverse events (SAE) according to the MedDRA classification. Some patients had more than one adverse event.

*patient reported moved to ICU.

No patient developed grade 4 neutropenia.

Incidence of hypersensitivity reactions [ Time Frame: Up to day 29 ]
There were two reported hypersensitivity reactions (injection reactions) in the anakinra arm and none in the tocilizumab arm. One patient in the UC arm developed an allergic reaction, two had skin rashes.

Incidence of infusion reactions [ Time Frame: Up to day 29 ]
There were no reported infusion reactions in the study.
