# Supplementary material for: Anakinra or tocilizumab in patients admitted to hospital with severe covid-19 at high risk of deterioration (IMMCoVA): A randomized, controlled, open-label trial
Source: PLoS One. 2023 Dec 29;18(12):e0295838. doi: 10.1371/journal.pone.0295838 (PMC10756513; doi:10.1371/journal.pone.0295838)
Supplement: S3 Table — (DOCX) [file pone.0295838.s003.docx]

**S3 Table. Adverse events by day 60**

| AE | UC (n=27) | | Anakinra (n=28) | | Tocilizumab (n=22) | |
| --- | --- | --- | --- | --- | --- | --- |
|  | Any grade | Severe (grade 1-2) , n (% of all AE) | Any grade, n (% of all AE) | Severe (grade 1-2) , n (% of all AE) | Any grade, n (% of all AE) | Severe (grade 1-2) , n (% of all AE) |
| Any adverse event | 68 | 29 | 62 | 11 | 13 | 6 |
| Any adverse event, no of patients | 17 | - | 21 | - | 9 | - |
| Angina Pectoris, n (% of total AE/group) | 1 (1.5) | 1(3.4) | 2 (3.2) | 0 | 0 | 0 |
| Arrythmia, n (%) | 0 | 0 | 1 (1.6) | 1 (9.1) | 0 | 0 |
| Cardiac dilatation, n (%) | 1 (1.5) | 1 (3.4) | 0 | 0 | 0 | 0 |
| Atrial fibrillation, n (%) | 2 (2.9) | 1 (3.4) | 0 | 0 | 0 | 0 |
| Tachycardia, n (%) | 1 (1.5) | 1 (3.4) | 2 (3.2) | 0 | 0 | 0 |
| Bradycardia, n (%) | 0 | 0 | 1 (1.6) | 0 | 0 | 0 |
| Heart failure, n (%) | 0 | 0 | 1 (1.6) | 1 (9.1) | 0 | 0 |
| Anemia, n (%) | 0 | 0 | 3 (4.8) | 2 (18.2) | 0 | 0 |
| Leukopenia, n (%) | 0 | 0 | 1 (1.6) | 0 | 0 | 0 |
| Thrombocytosis, n (%) | 0 | 0 | 1 (1.6) | 0 | 0 | 0 |
| Thrombocytopenia, n (%) | 0 | 0 | 1 (1.6) | 0 | 1 (7.7) | 0 |
| Tinnitus aggravated, n (%) | 0 | 0 | 1 (1.6) | 0 | 0 | 0 |
| Visual impairment, n (%) | 0 | 0 | 0 | 0 | 1 (7.7) | 1(16.7) |
| Diarrhea, n (%) | 1(1.5) | 0 | 3 (4.8) | 0 | 0 | 0 |
| Pancreatitis, n (%) | 1 (1.5) | 1 (3.4) | 0 | 0 | 0 | 0 |
| Gastrooesophageal reflux, n (%) | 1 (1.5) | 0 | 0 | 0 | 0 | 0 |
| Dyspepsia, n (%) | 0 | 0 | 1 (1.6) | 0 | 0 | 0 |
| Epigastric pain, n (%) | 0 | 0 | 1 (1.6) | 0 | 0 | 0 |
| Impaired gastric emptying, n (%) | 1 (1.5) | 1 (3.4) | 0 | 0 | 0 | 0 |
| Constipation, n (%) | 0 | 0 | 0 | 0 | 1 (7.7) | 0 |
| Hemorrhoids, n (%) | 0 | 0 | 0 | 0 | 1 (7.7) | 0 |
| Fever, n (%) | 6 (8.8) | 3 (10.3) | 1 (1.6) | 0 | 1 (7.7) | 1 (16.7) |
| Critical illness, n (%) | 1 (1.5) | 1 (3.4) | 0 | 0 | 0 | 0 |
| General body pain, n (%) | 1 (1.5) | 0 | 0 | 0 | 0 | 0 |
| Infusion site swelling, n (%) | 0 | 0 | 0 | 0 | 1 (7.7) | 1 (16.7) |
| Urinary tract infection, n (%) | 3 (4.4) | 1 (3.4) | 1 (1.6) | 0 | 1 (7.7) | 0 |
| Sepsis, n (%) | 1 (1.5) | 1 (3.4) | 0 | 0 | 0 | 0 |
| Clostridium difficile infection, n (%) | 0 | 0 | 1 (1.6) | 0 | 1 (7.7) | 0 |
| Candida infection, n (%) | 1 (1.5) | 0 | 0 | 0 | 0 | 0 |
| Candida sepsis, n (%) | 0 | 0 | 1 (1.6) | 1 (9.1) | 0 | 0 |
| Bacterial Pneumonia, n (%) | 0 | 0 | 1 (1.6) | 1 (9.1) | 0 | 0 |
| Giardiasis, n (%) | 1 (1.5) | 0 | 0 | 0 | 0 | 0 |
| Transaminases increased, n (%) | 1 (1.5) | 1 (3.4) | 4 (6.4) | 1 (9.1) | 1 (7.7) | 0 |
| Amylase increased, n (%) | 0 | 0 | 1 (1.6) | 0 | 0 | 0 |
| GT increased, n (%) | 0 | 0 | 1 (1.6) | 0 | 0 | 0 |
| Low potassium in serum, n (%) | 0 | 0 | 1 (1.6) | 0 | 0 | 0 |
| D-dimer increased, n (%) | 2 (2.9) | 1 (3.4) | 1 (1.6) | 0 | 0 | 0 |
| Low eGFR, n (%) | 0 | 0 | 2 (3.2) | 0 | 0 | 0 |
| Blood fibrinogen increased, n (%) | 0 | 0 | 2 (3.2) | 0 | 0 | 0 |
| Blood lactate acid abnormal, n (%) | 0 | 0 | 1 (1.6) | 0 | 0 | 0 |
| BNP increased, n (%) | 0 | 0 | 1 (1.6) | 0 | 0 | 0 |
| Fibrin increased, n (%) | 0 | 0 | 1 (1.6) | 0 | 0 | 0 |
| Hypernatremia, n (%) | 1 (1.5) | 0 | 0 | 0 | 0 | 0 |
| Hyperglycemia, n (%) | 0 | 0 | 1 (1.6) | 0 | 0 | 0 |
| Back pain, n (%) | 1 (1.5) | 1 (3.4) | 0 | 0 | 0 | 0 |
| Swelling of knee, n (%) | 0 | 0 | 1 (1.6) | 0 | 0 | 0 |
| Adrenal adenoma (detected) , n (%) | 0 | 0 | 1 (1.6) | 0 | 0 | 0 |
| Headache, n (%) | 1 (1.5) | 0 | 1 (1.6) | 0 | 0 | 0 |
| Paresthesias, n (%) | 1 (1.5) | 0 | 1 (1.6) | 0 | 0 | 0 |
| Hypotonia, n (%) | 1 (1.5) | 0 | 0 | 0 | 0 | 0 |
| Critical illness polyneuropathy and myopathy, n (%) | 2 (2.9) | 2 (6.9) | 0 | 0 | 0 | 0 |
| Dizziness, n (%) | 0 | 0 | 0 | 0 | 1 (7.7) | 1 (16.7) |
| Delirium (ICU) , n (%) | 1 (1.5) | 1 (3.4) | 0 | 0 | 0 | 0 |
| Irritability, n (%) | 0 | 0 | 1(1.6) | 0 | 0 | 0 |
| Panic attacks, n (%) | 0 | 0 | 1(1.6) | 0 | 0 | 0 |
| Renal failure, n (%) | 2 (2.9) | 2 (6.9) | 0 | 0 | 0 | 0 |
| Kidney stone, n (%) | 0 | 0 | 1(1.6) | 1 (9.1) | 0 | 0 |
| Vaginal prolapse, n (%) | 0 | 0 | 0 | 0 | 1 (7.7) | 1 (16.7) |
| Pulmonary embolism/microembolism, n (%) | 2 (2.9) | 1 (3.4) | 0 | 0 | 0 | 0 |
| Dyspnoea, n (%) | 2 (2.9) | 2 (6.9) | 0 | 0 | 0 | 0 |
| Nosebleed, n (%) | 2 (2.9) | 0 | 2 (3.2) | 0 | 0 | 0 |
| Tachypnea, n (%) | 1 (1.5) | 1 (3.4) | 0 | 0 | 0 | 0 |
| Pulmonary hypertension, n (%) | 1 (1.5) | 1 (3.4) | 0 | 0 | 0 | 0 |
| Exanthema, n (%) | 3 (4.4) | 1 (3.4) | 1 (1.6) | 0 | 0 | 0 |
| Urticaria, n (%) | 0 | 0 | 1 (1.6) | 0 | 0 | 0 |
| Hair loss, n (%) | 0 | 0 | 1 (1.6) | 0 | 0 | 0 |
| Hypertension, n (%) | 1 (1.5) | 0 | 0 | 0 | 0 | 0 |
| Hypotension, n (%) | 2 (2.9) | 0 | 3 (4.8) | 0 | 0 | 0 |
| Deep Vein Thrombosis, n (%) | 0 | 0 | 1 (1.6) | 1 (9.1) | 0 | 0 |
| Other* , n (%) | 19 (27.9) | 3 (10.3) | 6 (9.7) | 2 (18.2) | 2 (15.4) | 1 (16.7) |

Adverse events according to the MedDRA classification. Some patients had more than one adverse event.

Includes for UC: Enterococcus culture in faeces positive (1), Fungal culture in tracheostoma positive (1), Staphylococci culture pos tracheostoma (1), Streptococci culture pos tracheostoma (1), Staphylococci positive culture in blood (1), Unresponsive to verbal stimuli (3, same patient at different occasions), Progress of lung infiltrates on x-ray (1), Move to ICU/intubation (2), Temporary low oxygen (1), Noradrenaline administration (1), Extubation (1), insertion of rectal sond (1), Palliative care (1), Hemodialysis (3); for ANA: Desaturation at mobilization (1), Increase in oxygen need (1), NIV start (2, same patient, different occasions), sleep disturbance (1), Increase in CRP (1); For Toci: Staph aureus culture pos sputum (2)
