# Supplementary material for: Anakinra or tocilizumab in patients admitted to hospital with severe covid-19 at high risk of deterioration (IMMCoVA): A randomized, controlled, open-label trial
Source: PLoS One. 2023 Dec 29;18(12):e0295838. doi: 10.1371/journal.pone.0295838 (PMC10756513; doi:10.1371/journal.pone.0295838)
Supplement: S1 File — (PDF) [file pone.0295838.s005.pdf]

CLINICAL STUDY PROTOCOL

A single-center, randomized, open-label study in patients with COVID-19 and respiratory distress not requiring mechanical ventilation, to compare standard-of-care with anakinra and tocilizumab treatment

The Immunomodulation-CoV Assessment (ImmCoVA) study

Version: 4.0 May 27, 2020

Study design: Single-center, randomized, open-label, adaptive study with three treatment arms.

EudraCT: 2020-001748-24

Investigator initiated trial (IIT):

Principal Investigator: Jonas Sundén-Cullberg, MD, PhD

Co-Investigators: Piotr Nowak, MD, PhD
Jan Vesterbacka, MD, PhD
Jon Lampa, MD, PhD
Anders Sönnerborg, MD, PhD, Prof.

Sponsor: Karolinska University Hospital

Administrative information

Sponsor<br><br>Sponsor representative	Karolinska universitetssjukhuset,<br><br>Jon Lampa<br>ME Gastro, Hud, Reuma<br>Karolinska universitetssjukhuset<br>17176 Stockholm<br>Jon.lampa@sll.se
Principal Investigator	Jonas Sundén-Cullberg<br>Infektionskliniken<br>Karolinska universitetssjukhuset<br>14186 Stockholm<br>Jonas.sunden-cullberg@sll.se
Monitoring	Karolinska Trial Alliance,<br>KTA Support<br>Sabbatsbergs sjukhus<br>Dalagatan 9<br>11324 Stockholm<br>kta@karolinska.se

1 Signatures

The undersigned has read and understood the study protocol and certifies that it contains all essential information regarding conduct of the study.

The study will be conducted in accordance with this study protocol, the ethical principles stated in the Declaration of Helsinki and that comply with ICH-GCP and applicable national laws and regulations.

Principal Investigator
Jonas Sundén-Cullberg, MD, PhD
Infektionskliniken
Karolinska University Hospital

Signature Date

Sponsor
Jon Lampa, MD, PhD
ME Gastro, Dermatology, Rheumatology
Karolinska University Hospital

Signature Date

2 Brief Summary

The study is designed as a randomized, controlled, single-center open-label trial to compare standard-of-care (SOC) treatment with SOC + anakinra or SOC + tocilizumab treatment in hospitalized adult subjects who are diagnosed with severe COVID 19.

Arm A: Standard-of-care Treatment (SOC)
Arm B: Anakinra + SOC
Arm C: Tocilizumab + SOC.

All subjects will be treated with standard-of-care treatment and broad spectrum antibiotics initiated before or latest 24 hours after initiation of treatment with study drug.
Ceftriaxone 2 g x 1 i.v administration or Cefotaxime 1 g x 3
alternatively in PC allergic patients:
Clindamycine 600 mg x 3 i.v administration and Ciprofloxacin p/o 500 mg x 2.
Alternative antibiotics or antibiotic combinations, for example piperacillin/tazobactam or carbapenems are also accepted, when clinically indicated.

The primary follow up period of the study is 29 days.

Content

1 Signatures 3
2 Brief Summary 4
3 Abbreviations 8
4 Background 10
5 Study objective 11
6 Trial design 11
7 Treatment regimens 11
8 PICO 12
9 Study Population 12
10 Eligibility Criteria 13
10.1 Key Inclusion Criteria: 13
10.2 Key Exclusion Criteria: 13
10.3 Criteria for withdrawal 14
11 Randomization and assignment of Subject numbers 15
12 Study objectives 15
13 Efficacy variables 16
13.1 Primary outcome measures 16
13.2 Secondary Outcome Measures 16
13.3 Exploratory objectives / laboratory tests 19
14 Prior and concomitant therapy 19
15 Safety variables 19
16 Removal of subjects from therapy or assignments 19
17 Subject improved and therefore discharged from hospital care: 20
18 Statistical analysis plan 21
18.1 Determination of sample size 21
18.2 Definition of study populations 21
18.3 Analysis related to primary objective and primary endpoint 22
18.4 Analysis related to secondary endpoints 22
18.5 Sub-group and adjusted analysis 22
18.6 Final visit of these subjects: 23
19 Interventions 23
19.1 Storage and Disposition of Study Drug(s) 24
19.2 Method of Assigning Subjects to Treatment Groups 24

19.3 Selection and Timing of Dose for Each Subject 24
19.4 Blinding 24
19.5 Treatment Compliance 24
20 Appropriateness of Measurements 25
21 Suitability of Subject Population 25
22 Study activities 25
22.1 From day 1 until day 15 25
22.2 From day 16 to day 60 26
23 Study Procedures 27
23.1 Inclusion/Exclusion criteria 27
23.2 Informed Consent 27
23.3 Medical History 27
23.4 Blood pressure, heart rate, respiratory frequency, Body temperature 28
23.5 Sequential organ failure assessment (SOFA) 28
23.6 The National Early Warning Score 2 (NEWS2) 28
23.7 SARS-CoV-2 Test 28
23.8 SARS-CoV-2 levels 28
23.9 Pregnancy test 28
23.10 Laboratory tests 28
23.11 Clinical Laboratory tests 28
24 End of Study 29
25 Adverse events 29
25.1 Serious Adverse event 30
25.2 Adverse Event Severity 30
25.3 Relationship to Study Drug 30
25.4 Adverse Event Collection Period 31
25.5 Serious Adverse Drug Reaction Reporting 31
26 Risk Benefit justification 31
27 Protocol Deviations 32
28 Coding and tabulation of Adverse Events 32
29 Independent Ethics Committee (IEC) 32
30 Ethical Conduct of the Study 32
31 Subject Information and Consent 33
32 Source Documents 33
33 Case Report Forms 33

34 Monitoring ... 33
35 Archiving ... 34
36 References ... 35

3 Abbreviations

Abbreviation	Explanation
SOC	Standard of Care
SARS-CoV-2	Severe acute respiratory syndrome Coronavirus 2
ARDS	acute respiratory distress syndrome
ICU	Intensive Care Unit
MERS	Middle East Respiratory Syndrome
CRS	cytokine releasing syndrome
HLH	hemophagocytic lymphohistiocytosis
MAS-HLH	macrophage activation syndrome
IL-	Interleukin
IL-1Ra	Interleukin 1 receptor a
sIL-6R	soluble IL-6 receptors
mIL-6R	membrane-bound IL-6 receptors
TNF	Tumor Necrosis Factor
SpO2	peripheral capillary oxygen saturation
FiO2	Fraction of inspired oxygen
I.v	Intravenously
NaCl	Sodium Chloride
PC	Penicillin
PCR	polymerase chain reaction
CRP	c-reactive protein
FSH	Follicle stimulating hormone
NYHA	New York Heart Association
BP	Blood pressure
ANC	absolute neutrophil count
AST	aspartate aminotransferase
ALT	alanine aminotransferase
eGFR	Estimated glomerular filtration rate
ULN	upper limit of normal
JAKi	Janus kinase inhibitors
DMARD	disease-modifying antirheumatic drugs
TB	Tuberculosis
IP	Investigational Product
ECMO	Extracorporeal membrane oxygenation
SOFA	sequential organ failure assessment score
NEWS	National Early Warning Score
HR	Heart Rate
SPSS	Statistical Package for the Social Sciences
ITT	intent-to-treat
BL	Baseline

IQR	interquartile range
RR	Relative Risk
GEE	Generalized Estimating Equation
JIA	Juvenile idiopathic arthritis
SC	Sub-cutaneous
SIRS	Systemic inflammatory response syndrome
ET	Early Termination
PI	Principle Investigator
NPH	nasopharynx
CSCT	Central Study Coordinating Team
IEC	Independent Ethics Committee
IRB	Independent Review Board
GCP	Good Clinical Practice
AE	Adverse Event
CRF	Case Report Form
SmPC	Summary of Product Characteristics
ICH	International Conference of Harmonisation
MPA	Medicinal Product Agency
SADR	Serious Adverse Drug Reaction
SAE	Serious Adverse Event
SUSAR	Suspected Unexpected Serious Adverse Reaction

4 Background

COVID-19 is a massive threat to public health worldwide[1]. As of March 18, 2020, cases were reported in approximately 195 countries[2]. The disease diagnosed COVID-19, caused by Severe acute respiratory syndrome Coronavirus 2 (SARS-CoV-2), with lung involvement exhibits features that are unlikely ameliorated by antivirals-based approaches alone. The antiviral efficacy of antiviral drugs under investigation, such as remdesivir and favipiravir, is not yet known[3,4].

Accumulating evidence shows that patients with severe COVID-19 have elevation of several pro-inflammatory cytokines[5], suggesting the importance of a cytokine releasing syndrome (cytokine storm) in the pathogenesis of the disease. Interventions aiming at decreasing hyperinflammation might therefore be beneficial in preventing acute respiratory distress syndrome (ARDS) and ICU admittance[6]. Notably, SARS-CoV-2, SARS-CoV and Middle East Respiratory Syndrome (MERS) CoV are coronaviruses, and cytokine releasing syndrome (CRS) of varying degrees has occurred in severely ill patients with SARS[7-10] and MERS[11]. Other conditions characterized by massive hyperinflammation as a prominent feature are hemophagocytic lymphohistiocytosis (HLH) and macrophage activation syndrome (MAS-HLH)[12]. These conditions are also characterized by high fever, hepatosplenomegaly, liver, central nervous system and kidney involvement, and may lead to multiple organ failure[13]. Interestingly, laboratory abnormalities in HLH and MAS-HLH include decrease in white blood cells, platelets and hemoglobin, elevated transaminases, marked increase in ferritin, and evidence of intravascular coagulation activation. Thus, these conditions share many features with the hyperinflammation and pattern of laboratory abnormalities described in COVID-19[5,10].

Interleukin 1 beta (IL-1beta) is an important cytokine in the context of hyperinflammation of MAS-HLH[12]. Moreover, IL-1 receptor blocker anakinra has been shown to efficiently decrease hyperinflammation in MAS-HLH[14]. Relating to the similarities in the cytokine pattern of MAS-HLH and COVID-19 with CRS, this indicates a potential for beneficial effects of anakinra in COVID-19 infection, and intervening with key cytokines of CRS, such as IL-1beta and interleukin 6 (IL-6) has been suggested[6]. However, there have also been discussions on the potential risks of administering cytokine blockers in conditions associated with CRS. In sepsis, CRS is known to be complicated by immune suppression[15] and interventions aiming at decreasing CRS may therefore further deteriorate the condition. Earlier reports have shown conflicting results, some with aggravation of the sepsis[16-18] but there have also been reports that anakinra may improve sepsis[19]. Thus, the results of treatment during septicaemia are conflicting, and an activation of the bacterial infection could not be excluded. It needs to be noted, however, that severe infections during treatment with cytokine blockers for CRS in MAS-HLH has not been a significant clinical problem[19].

IL-6 is an important member of the cytokine network and plays a central role in acute inflammation[20]. Tocilizumab is a recombinant humanized monoclonal antibody against human IL-6 receptor of immunoglobulin IgG1 subtype. Tocilizumab specifically binds soluble and membrane-bound IL-6 receptors (sIL-6R and mIL-6R) and inhibits sIL-6R and mIL-6R-mediated signal transduction. Tocilizumab is approved for the treatment of rheumatoid arthritis[21] and systemic juvenile idiopathic arthritis[22]. The main side effects of tocilizumab are elevated transaminases, leukopenia and hypercholesterolemia. In similarity to other biologics, such as TNF-blockers, tocilizumab has been shown to be associated with increased risk of severe bacterial infections[23,24].

Notably, data from the earlier reports on CRS associated with SARS and MERS revealed that all patients had high expression of IL-6[7,11]. Moreover, tocilizumab was shown effective in the treatment of severe CRS patients[25] and is approved in Sweden for treatment of Car T cell related cytokine storm.

Off-label use in COVID-19 has potentially been associated with beneficial effects in a number of patients worldwide and a recent small single-arm study indicated beneficial effects of IL-6 blockade on COVID-19 with respiratory distress[26].

Interim analysis of a recent observational case-control trial (the SISCO study) indicated that 76% of the patients with COVID-19 and respiratory distress improved or remained stable 7 days after a single infusion with the IL-6 blocker siltuximab[27]. And interim results from an RCT of another IL-6 receptor antibody, sarilumab, indicated lower incidence of the combined endpoint of mechanical ventilation and/or death in critically ill patients in those on the higher dose of sarilumab, compared to placebo, 32 vs 55%.

5 Study objective

To assess and compare standard-of-care (SOC) treatment only to a combination of SOC and IL-1 receptor antagonist anakinra or IL-6 receptor blocker tocilizumab concerning effects on time to recovery in hospital admitted patients with COVID-19 infection, respiratory distress and increased risk of clinical deterioration.

6 Trial design

All patients will receive the updated SOC treatment protocol according to local recommendations at the Karolinska University Hospital.

40 patients will receive SOC treatment and no cytokine-blocker (arm A).**40** patients will be treated with SOC and anakinra (Kineret[R]) (arm B), **40** patients will be treated with SOC and tocilizumab (RoActemra[R]) (arm C).

The study design is a randomized, controlled, open-label, adaptive trial with three treatment arms.

The adaptive design will allow for increasing the sample size, provided this is suggested at a blinded interim analysis, performed after inclusion of 50% of the study patients.

7 Treatment regimens

Arm A: Standard-of-care treatment (SOC treatment)
Oxygen supplementation so as to achieve SpO2 ≥93 %, antipyretic treatment (paracetamol) and thrombosis prophylaxis (Fragmin® or Innohep®) to the immobilized patients.

The original protocol recommends that antibiotics should only be used in patients with confirmed bacterial coinfection or in patients with high clinical suspicion of bacterial coinfection. However,

due to the nature of this study and the potential of aggravation of bacterial infections during cytokine blockade (see above) all patients will receive broad spectrum antibiotics, see below.

Arm B: Anakinra (Kineret^R)
(Kineret^R) a total of 400 mg i.v / day given every 6 hours* (i.e. 100 mg x 4 i.v.) for 7 days.
This treatment will be combined with SOC treatment.

*Anakinra may NOT be administered concomitantly via y-site or mixed with any other medications due to no compatibility information. Each injection is followed by a flush of NaCL.

Arm C: Tocilizumab (RoActemra^R)
RoActemra^R 8 mg/kg for a single infusion i.v. up to max 800 mg.
1 more infusion (8 mg/kg) may be administered earliest after 2 days with the following condition: The clinical symptoms are worsened (as assessed by decreasing PaO2/FiO2 and/or need of increased ventilatory support such as NIV, HFNC or mechanical ventilation).
This treatment will be combined with SOC treatment.

Additional treatment for all patients (arm A, B and C):
All patients in all arms will be treated with prophylactic broad spectrum antibiotics initiated before or latest 24 hours after initiation of treatment with study drug for seven days. May be prolonged if clinically indicated.

Ceftriaxone 2 g x 1 i.v administration or Cefotaxime 1 g x 3
Alternative in PC allergic patients: Clindamycine 600 mg x 3 i.v administration and T. Ciprofloxacin 500 mg x 2. Alternative antibiotics or antibiotic combinations, for example piperacillin/tazobactam or carbapenems are also accepted, when clinically indicated.

8 PICO

The study will include critically ill, and respiratory distressed patients with COVID-19 infection **(Population)**, treated with anakinra (IL-1Ra) or tocilizumab (anti-IL-6R) **(Intervention)** compared to standard-of-care treatment **(Control)** to decrease time to recovery **(Primary Outcome)** and to decrease disease severity and time in mechanical ventilation and improve oxygenation **(secondary outcomes)**.

9 Study Population

Ages Eligible: 18-80 Years (Adult, Older Adult)

Sexes Eligible: All (Male, Female, Transgender)

10 Eligibility Criteria

10.1 Key Inclusion Criteria:

1) Laboratory-confirmed SARS-CoV-2 infection as determined by polymerase chain reaction (PCR) or other commercial or public health assay
2) SARS-CoV-2 infection with duration at least 7 days (as determined by onset of symptoms)
3) PaO2 (or SpO2)/FiO2 < 26,8 kPa (200 mm Hg) for at least 8 hours, corresponding to 5 liters/minute of Oxygen to maintain SpO2 at 94%.
4) CRP > 70 mg/L with no non-SARS-Cov2 infections.
5) Ferritin > 500 µg/L
6) At least two points on a scale of 0-3 where 1 point is awarded for each value of; lymphocytes < 1x 10(9)/L; D-dimer ≥ 0.5 mg/L and; Lactate Dehydrogenase ≥ 8 microkatal/L.
7) Ability to provide informed consent signed by study patient
8) Willingness and ability to comply with study-related procedures/assessments
9) In fertile females, willing to comply with effective contraceptive methods for up to 3 months after last dose of study drug. These may include birth control pills, surgical sterilization of patient or partner or intrauterine device. Non-fertile woman is defined as more than 12 months of amenorrhea without an alternative medical cause or, in case of ambiguities, an FSH level in the postmenopausal range.

10.2 Key Exclusion Criteria:

1) Pregnancy or breast feeding.
2) Ongoing or completed mechanical ventilation.
3) In the opinion of the investigator, unlikely to survive for >48 hours from screening.
4) In the opinion of the investigator, expected overall survival due to other comorbidities less than 3 months.
5) Chronic impairment of cardiac function NYHA II or higher.
6) Severe renal dysfunction eGFR < 30 ml/min.
7) Medical history including chronic liver disease with inflammation, fibrosis or cirrhosis including underlying diseases such as alcoholic liver disease, non-alcoholic fatty liver disease, chronic viral hepatitis, alcoholic liver disease, autoimmune liver disease, hemochromatosis, Wilson’s disease, alpha-1 antitrypsin deficiency, cholangitis, or carcinoma.
8) Uncontrolled hypertension Systolic BP >180 mm Hg, Diastolic BP > 110 mm Hg
9) History of hypersensitivity to the study drugs
10) Presence of any of the following abnormal laboratory values at screening: absolute neutrophil count (ANC) less than 2 x 10^9/L, aspartate aminotransferase (AST) or alanine aminotransferase (ALT) greater than 5 x upper limit of normal (ULN), platelets <100 x 10^9/L
11) Treatment with anakinra, anti-IL 6, anti-IL-6R antagonists, Janus kinase inhibitors (JAKi) in the past 30 days or plans to receive during the study period

12) Current treatment with conventional synthetic disease-modifying antirheumatic drugs (DMARDs)/immunosuppressive agents
13) Use of chronic oral corticosteroids for a non-COVID-19-related condition in a dose higher than prednisone 10 mg or equivalent per day
14) History of, or current autoimmune or inflammatory systemic or localized disease(s) other than rheumatoid arthritis
15) Acute systemic infection; verified by blood cultures systemic bacterial infection, systemic fungi-infection or prosthesis-related infection
16) History of stem-cell or solid organ transplantation
17) Known active tuberculosis (TB), history of incompletely treated TB, suspected or known extrapulmonary TB, suspected or known systemic bacterial or fungal infections
18) Diagnosis of, or suspicion of HIV infection, acute hepatitis A and/or chronic hepatitis B and/or C
19) Previous history of gastrointestinal ulceration or diverticulitis.
20) Patients who have received immunosuppressive antibody therapy within the past 3 months, including intravenous immunoglobulin or plans to receive during the study period
21) Participation in any clinical research study evaluating an investigational product (IP) or therapy within 3 months and less than 5 half-lives of IP prior to the screening visit. The use of remdesivir in the context of a single-arm remdesivir compassionate use protocol is permitted)
22) Any physical examination findings and/or history of any illness that, in the opinion of the study investigator, might confound the results of the study or pose an additional risk to the patient by their participation in the study

10.3 Criteria for withdrawal

Patients are free to discontinue their participation in the study at any time for any reason without affecting their right to appropriate follow-up investigation and future treatment.

Patients may be discontinued from the study at any time at the discretion of the Investigator. Specific reasons for discontinuing a patient from further assessments are:

• Withdrawal of informed consent.

• If the Investigator considers it not to be in the patient´s best interest to continue participation in the study.

• If there is a significant protocol deviation.

• If a concomitant therapy is reported or required which is likely to interfere with the results of the study or compromise subject safety.

To assess any potential impact on subject eligibility with regard to safety, the investigator must refer to the following document for detailed information regarding warnings, precautions, contraindications, adverse events (AEs) and other significant data pertaining to Kineret® or Roactemra®: Approved national summary of product characteristics (SmPC).

Final assessments should be performed if possible. The reasons for a subject having discontinued the study will be documented in the Case Report Form (CRF). If a subject discontinues from the study due to an AE, the nature of the event and its clinical course must be fully documented. The investigator must follow the subject until the AE has resolved, become clinically insignificant, or is stabilized, unless the subject is lost to follow-up. If a subject is lost to follow up the Investigator must make every effort to contact the subject. This will be recorded in the CRF.

11 Randomization and assignment of Subject numbers

Subjects who meet the selection criteria will proceed to randomization. Stratification will be done by age and gender. Age will be categorized according to:
1. 18-49
2. 50-69
3. 70-80

Gender will be categorized according to (based on the assumed low prevalence of Females and Transgender)

1. Male
2. Female and Transgender

Randomization will be performed using block-randomization.

12 Study objectives

Primary objective
The primary objective is to assess the effect of anakinra and tocilizumab on time to recovery in patients with COVID-19 and respiratory distress.

Secondary objective
Secondary objectives of the study are to assess the effect of anakinra and tocilizumab on mortality, need and length of intensive care, and also rate, magnitude and speed of clinical improvement including pulmonary function in patients with COVID-19 and respiratory distress.

Exploratory objective(s)
The exploratory objective of the study is to assess the effect of anakinra and tocilizumab on selected biomarkers relevant for hyperinflammation and coagulation disturbances.

13 Efficacy variables

13.1 Primary outcome measures

1. Time to recovery [Time Frame: Day 1 through Day 29]

Day of recovery is defined as the first day on which the subject satisfies one of the following three categories from the ordinal scale:
1) Hospitalized, not requiring supplemental oxygen - no longer requires ongoing medical care [1];
2) Not hospitalized, limitation on activities and/or requiring home oxygen;
3) Not hospitalized, no limitations on activities.

[1] LMWH-injections (Fragmin, Innohep) do not count as medical care

13.2 Secondary Outcome Measures

1. Mortality [Time Frame: Up to day 29]

2. Number of Days on mechanical ventilation [Time Frame: Up to day 29]

3. Number of days of supplemental oxygen use [Time Frame: Up to day 29]

4. Number of patients requiring initiation of mechanical ventilation [Time Frame: Up to day 29]

5. Time to improvement in oxygenation for at least 48 hours [Time Frame: Up to day 29]
   Definition of improvement in oxygenation: Increase in SpO2/FiO2 of 50 or greater compared to the nadir SpO2/FiO2

6. Mean change in the 8-point ordinal scale [Time Frame: Up to day 29]

   8-point Ordinal Scale:

   a) Death
   b) Hospitalized, on invasive mechanical ventilation or ECMO;
   c) Hospitalized, on non-invasive ventilation or high flow nasal cannula;
   d) Hospitalized, requiring supplemental oxygen
   e) Hospitalized, not requiring supplemental oxygen
   f) Hospitalized, not requiring supplemental oxygen - no longer requires ongoing medical care
   g) Not hospitalized, limitation on activities and/or requiring home oxygen;
   h) Not hospitalized

7. Proportion of patients on level e-h on the 8-point ordinal scale at day 15

8-point Ordinal Scale:

a) Death
b) Hospitalized, on invasive mechanical ventilation or ECMO;
c) Hospitalized, on non-invasive ventilation or high flow nasal cannula;
d) Hospitalized, requiring supplemental oxygen
e) Hospitalized, not requiring supplemental oxygen
f) Hospitalized, not requiring supplemental oxygen - no longer requires ongoing medical care
g) Not hospitalized, limitation on activities and/or requiring home oxygen;
h) Not hospitalized

8. Time to improvement in one category from admission using the 8-point ordinal scale [Time Frame: Up to day 29]

   8-point Ordinal Scale:

   a) Death
   b) Hospitalized, on invasive mechanical ventilation or ECMO;
   c) Hospitalized, on non-invasive ventilation or high flow nasal cannula;
   d) Hospitalized, requiring supplemental oxygen
   e) Hospitalized, not requiring supplemental oxygen
   f) Hospitalized, not requiring supplemental oxygen - no longer requires ongoing medical care
   g) Not hospitalized, limitation on activities and/or requiring home oxygen;
   h) Not hospitalized

9. Mean change in SOFA score [Time Frame: Up to day 29]

10. Time to resolution of fever for at least 48 hours by clinical severity [Time Frame: Up to day 29]

    Defined as ≤36.6°C (axilla), ≤37.2°C (oral) or ≤37.8°C (rectal or tympanic)

11. Time to change in National Early Warning Score 2 (NEWS2) scoring system [Time Frame: Up to day 29]

    NEWS2 consists of: Physiological Parameters: Respiration rate (per minute), SpO2 Scale 1 (%), SpO2 Scale 2 (%), Use of air or oxygen, Systolic blood pressure (mmHg), Pulse (per minute), Consciousness, Temperature (°C)

12. Time to score of <2 maintained for 24 hours in NEWS2 scoring system [Time Frame: Up to day 29]

    NEWS2 consists of: Physiological Parameters: Respiration rate (per minute), SpO2 Scale 1 (%), SpO2 Scale 2 (%), Use of Air or oxygen, Systolic blood pressure (mmHg), Pulse (per minute), Consciousness, Temperature (°C)

13. Mean change in NEWS2 scoring system [Time Frame: Up to day 29]

14. Number of days with fever [Time Frame: Up to day 29]

    Defined as >36.6°C (axilla), >37.2°C (oral) or >37.8°C (rectal or tympanic)

15. Number of days of resting respiratory rate >24 breaths/min [Time Frame: Up to day 29]

16. Time to saturation ≥94% on room air [Time Frame: Up to day 29]

17. Incidence of serious adverse events [Time Frame: Up to day 60]

18. Incidence of severe or life-threatening bacterial, invasive fungal, or opportunistic infection [Time Frame: Up to day 29]

19. Incidence of severe or life-threatening bacterial, invasive fungal, or opportunistic infection in patients with grade 4 neutropenia [Time Frame: Up to day 60]

20. Incidence of hypersensitivity reactions [Time Frame: Up to day 29]

21. Incidence of infusion reactions [Time Frame: Up to day 29]

22. Number of ventilator free days in the first 28 days [Time Frame: Baseline to day 29]

23. Number of patients requiring non-invasive ventilation [Time Frame: Up to day 29]

24. Number of patients requiring the use of high flow nasal cannula [Time Frame: Up to day 29]

25. Number of patients requiring ECMO [Time Frame: Up to day 29]

26. Number of patients that have been admitted into an intensive care unit (ICU) [Time Frame: Up to day 29]

27. Number of days of hospitalization in survivors [Time Frame: Up to day 29]

28. Number of deaths due to any cause [Time Frame: Up to day 60]

13.3 Exploratory objectives / laboratory tests

Blood culture for bacteria/fungi. Nasopharyngeal and/or sputum culture: Performed before inclusion or (if not already performed) latest at inclusion/randomization.
Serum/plasma samples. PaxGene tubes for mRNA-seq. Baseline (= At start of treatment) and at five time points thereafter, see item 22.1.

For a smaller number of patients (10 pts from group A, B and C respectively, in total 30 patients): 10 ml Heparinized blood for ficoll separation and freezing cells in DMSO.

Suggestions of other biomarkers (analyzed later):
IL-1β, Il-18/IL-18 BP, IL-6 receptor, sIL-2 receptor, IL-2, TNF-alpha, CXCL9, CXCL10, IFN-gamma, IL-8, IL-10, TGF-beta

CYTOF (PaxGene tubes)

Samples for Global hemostasis (for ex thrombine induction, overall haemostatic potential, fibrin networks, PAI-1), Angiotensin-2

Functional analyses with frozen cells for B-cell immunity against the virus, and follow-up of the CYTOF results.

14 Prior and concomitant therapy

Any medication (including, but not limited to, over-the-counter medicines such as aspirin, antacids, vitamins, mineral supplements and herbal preparations) that the subject received 28 days prior to Screening, must be recorded on the source documents and on the appropriate page of the CRF along with the reason for use, dates of administration and dosages.
All concomitant medications and treatments are to be recorded in the appropriate CRF.

15 Safety variables

Safety will be assessed by AEs, physical examination, vital signs and laboratory data

16 Removal of subjects from therapy or assignments

Assigned therapy will be discontinued if any of the following occur: anaphylactic reactions, angioedema, urticaria or rash.

Clinically significant abnormal laboratory results which rule out continuation of the study medication, as determined by the investigator.

Abnormal laboratory results leading to discontinuation of therapy include, but are not limited to, changes in absolute neutrophil count, platelet count, AST, ALT and eGFR as detailed below:

A second dose of Tocilizumab is ruled out if;
Absolute neutrophil count drops below 0.5 x 10^9/L
Platelet count drops below 50 x 10^9/L
Aspartate aminotransferase (AST) or alanine aminotransferase (ALT) rises to greater than 5 x upper limit of normal (ULN)

Treatment with Anakinra is stopped if;
Absolute neutrophil count drops below 1.5 x 10^9/L
Platelet count drops below 100 x 10^9/L
Patient develops severe renal dysfunction, eGFR < 30 ml/min.(If eGFR is 30 to 59 ml/min; dose is adjusted, see section 22).

Other AEs which rule out continuation of the study medication, as determined by the investigator.

17 Subject improved and therefore discharged from hospital care:

In this study, the subject can be discharged from the hospital earliest day 7 after study start. Subjects that are discharged from hospital (due to improvement and no need of oxygen) will perform the following procedures:

Subject discharged after day 7, but before day 10:
Subject will not undergo measurements of the following procedures for the rest of the days until day 14: Body temperature, BP, HR, Respiratory Frequency, PO2/FiO2, NEWS2.

However, it is mandatory that the subject comes back day 10 for the following assessments: SOFA, CRP, Ferritin, Routine clinical samples including ALT, Research samples / biobanking

Subject discharged after day 10 but before day 15:
Subject will not undergo measurements of the following procedures the rest of the days until day 14: Body temperature, BP, HR, Respiratory Frequency, PO2/FiO2, NEWS2.

Final visit of these subjects:
It is mandatory that the subject return day 15 and day 29 for the final assessments according to the protocol. All subjects will have a follow up visit on Day 60, phone call and/or medical records, to assess SAEs.

18 Statistical analysis plan

18.1 Determination of sample size

The sample size has been calculated based on the conventional "fixed approach" and also considering the option of adaptively changing the design using the emerging treatment difference[28]. Thus calculation of the sample size was based on two approaches: the classical conventional approach and the adaptive design approach[29] (the sample size will be re-estimated based on the findings of the interim analysis).

Related to clinical experience, and from pre-trial data the SOC treatment group can be expected to reach the primary outcome after a mean of 15 +/- 6 days. Interim analysis of the SISCO study and previous off-label experience in Karolinska University Hospital of tocilizumab suggest that the time for reaching the primary outcome can be decreased to 11 days.

Taking the significance level at 5% and Type II error as 20% and considering the mean difference to be 4 days, 36 subjects are required per group to obtain 80% power. Given an anticipated dropout rate of 10 %, a total of 40 subjects per group will be included in the study to obtain 80% power.

Since the available data on covid-19 and potential effects of interventions to date is sparse, there is a risk that small variations in the assumed magnitude of difference in reaching the primary outcome in the control group may result in decreased power of the analysis. Therefore, an adaptive design has been introduced, allowing re-estimation of sample size at an interim point, as described[30]. Considering this adaptive design and assuming a mean difference for 4 days (see above)- the study will start with 36 subjects per group (80% power). Then at an interim point when about 50% of the patients have been included, analysis will be performed and the result will be observed and a blinded sample size re-estimation will be calculated. Analysis will be performed by a statistician not involved in the conduct of the trial, unaware of group allocation.Then the final sample size will be adapted to that interim result.Hence, after interim analyses the study may continue as planned 40 subjects will be included considering for a 10% drop out rate, or increase the sample size based on the interim findings or stop for futility.

All statistical analyses will be done using the Statistical Package for the Social Sciences (SPSS) for Windows (version 25; IBM, NY, USA) with the level of significance set at 0.05 (two-sided analysis). A professional academic statistician will conduct all analyses.

18.2 Definition of study populations

All measurements collected in this study will be analyzed based on intent-to-treat (ITT) principle. The ITT population will be defined as all subjects that initiated the study and study procedures after completing the informed consent. Demographic information and BL characteristics of these subjects will be summarized by count and percentages for discrete measurements and by summary statistics (mean, standard deviation *etc.*) for continuous measurements.

18.3 Analysis related to primary objective and primary endpoint

Descriptive statistics will be presented as means (standard deviations), and median (IQR) for numerical variables, or as frequencies or percentages for categorical variables. The main analysis will be conducted as an intention to treat (ITT) analysis for all patients that are randomly assigned in the study. Time to clinical improvement/deterioration will be assessed after all patients reach day 29; no clinical improvement at day 29 or death before day 29 will be considered as right censored at day 29. Kaplan-Meier plot will be used to plot the time to clinical improvement and a log-rank test will be performed to compare the groups. Cox proportional hazards model will be employed and the hazard ratio for clinical improvement with 95% CI reported. Adjusted analysis will be used to control for baseline variables (for example age and sex) and adjust for potential predictor variables which might reasonably be expected to be related to the primary outcome variable. Sensitivity analyses will be performed using the per protocol analysis. Multicollinearity and outlier problems will be checked. A Variance Inflation Factor (VIF) greater than 5 will be considered a cut-off criterion for deciding when a given independent variable displayed "too great" a multicollinearity problem. A Cook's distance value of (D) > 1 will be taken as a criterion to constitute a strong indication of outlier problems and $D > 4/n$ a criterion to indicate a possible problem, where n is the sample size.

18.4 Analysis related to secondary endpoints

For secondary outcome variables two-sample t test or Mann-Whitney, as appropriate, will be employed for continuous variables, Fischer's exact test for categorical variables. Levine's test will be used to check the assumption of equality of variance for the t-test.

18.5 Sub-group and adjusted analysis

Additional analyses will be carried out for the primary outcome variable. First, an adjusted analysis will be used to control for baseline variables. Secondly, a subgroup analysis will be performed to detect differences between groups of patients considering variables age group and gender. For subgroup analyses, we will use regression methods with appropriate interaction terms (respective subgroup × treatment group). Poisson regression will be used for count variables and Kaplan-Meier plot (compared with log rank test) followed by multivariable Cox proportional hazards model for time to event data.

For evaluating effect size, the difference between means or proportions, RR, 95% confidence intervals, partial eta-squared (η^2), and R^2 will be used. Residual plots, normal probability plots, and Cook's distance will be used to assess model assumptions. A Cook's distance (D) > 1 provided a strong indication of outlier problems, and D > 4/n, where n is the sample size, indicated a possible problem. A Variation Inflation Factor (VIF) greater than 5 is the cut-off criterion for deciding when a given independent variable displayed too great of a multicollinearity problem. We will analyze the data using intention to treat analysis for the primary outcome and separate testing with multiplicity adjustment for secondary outcomes.

18.6 Final visit of these subjects:

Repeated measurement analysis will be performed in subjects for comparison baseline, day 15 and day 29 (for the final assessments) according to the protocol. SAEs will also be followed up on Day 60.

Linear mixed model will be used to account for repeated measures, to model within-subject variance, and to handle correlated data of continuous variables. For binary and ordinal outcome variables Generalized Estimating Equation (GEE) will be employed. An interaction term will be introduced in the model to examine heterogeneity effect. The projected sample size will provide sufficient statistical power for linear mixed regression and GEE models considering compound symmetry of the covariance structure and the number of repeated measures. For the main analysis no missing data will be imputed. However, classical multiple imputation methods will be used for an additional sensitivity analysis if any of the included variables has more than 5% missing observations. The GEE is a technique which produces unbiased estimates under the assumption that missing observations will be missing at random. An amended approach of weighted GEE will be employed if missingness is found not to be at random. We will perform residual analysis to assess model assumptions and goodness-of-fit.

Adverse events (AEs) will be summarized by number and percentage of subjects experiencing the AE; the rate of AEs normalized by the study drug exposure will also be provided. Laboratory measurements and vital sign measurements will be summarized by summary statistics and shift tables as appropriate.

19 Interventions

All study medications are approved and established agents for which detailed information is available at all relevant national medicine product agencies.
Treatment schedule

Arm	IMP	Route	Daily Dose	Dosage regimen
A	Standard of Care	N/A	N/A	N/A
B	Anakinra	i.v.	A total of 400 mg/day, given every 6 hours	4 times daily for 7 days
C	RoActemra	i.v	8 mg/kg day 1<br>8 mg/kg earliest day 2	1 x 1 at inclusion<br>Another dose earliest day 2 provided fulfillment of specific criteria*

*The clinical symptoms are worsened (as assessed by decreasing PaO2/FiO2 and/or need of increased ventilatory support such as NIV, HFNC or mechanical ventilation).

19.1 Storage and Disposition of Study Drug(s)

Biologicals provided by the pharmacy as pre-filled syringes are to be stored according to product-specific information; usually meaning protected from light at 2°C-8°C/ 36°F-46°F, and not be frozen at any time.

19.2 Method of Assigning Subjects to Treatment Groups

All subjects will be centrally randomized.

19.3 Selection and Timing of Dose for Each Subject

All drugs will be administered by the clinical personnel in Karolinska University Hospital.

Anakinra: It should be noted that the usual recommended dose of anakinra (usually between 1 and 2 mg/kg/d) is for patients with JIA and is not necessarily applicable in this setting. Here, a higher than commonly recommended doses of anakinra is used for several reasons. First, the half-life of anakinra SC is very short (4–6 hr), justifying more frequent than daily dosing in the setting of acute systemic inflammation, in order to rapidly achieve therapeutic IL-1 inhibition. Moreover, there is published experience using high doses of continuous IV IL-1 receptor antagonist (IL-1ra) in sepsis patients, providing support for the safety of higher doses in an SIRS setting, even without evidence of any compelling therapeutic effect in that analysis[30]. Moreover, a study in children with systemic onset juvenile arthritis complicated by refractory macrophage activation syndrome is currently ongoing (NCT02780583). In this study, anakinra is administered at dose of 10 mg/kg/day to a maximum of dose of 200 mg/day divided every 12 hours (for children ≤40 kg) or 5 mg/kg/day up to a maximum dose of 400 mg/day divided every 6 hours (children > 40 kg and adults).
Based on the above, anakinra will in this study be administered with a total dose of 400 mg per day (divided in 4 doses of 100 mg i.v. every 6 hours).
Dose reduction in patients with renal dysfunction: In patients having a CL_{cr} 30 to 59 ml/min; dose adjustment of anakinra will be: 400 mg i.v. every other day.

Tocilizumab: The dose used in this study is in accordance with the recommended dose for rheumatoid arthritis, i.e. 8 mg/kg for a single infusion i.v. up to max 800 mg. In accordance with the recommended dose in cytokine releasing syndrome, another dose of 8 mg/kg may be administered if no clinical response is obtained. The original recommendation (for Car T cell syndrome) is another dose after 12 hours. However, in this protocol the clinical response is not evaluable at that time point, so the additional dose will be administered earliest after 2 days.

19.4 Blinding

This is an open-label study. The investigator, staff, and patient will be aware of treatment assignment. The analyses of data will be done by a blinded assessor.

19.5 Treatment Compliance

Since the study will be performed on critically ill patients, the investigator or his/her designated representatives will administer/dispense study drugs.

20 Appropriateness of Measurements

All efficacy measurements in this study have been published and used in the evaluation of respiratory distress. All clinical and laboratory procedures in this study are standard and generally accepted.

21 Suitability of Subject Population

Subjects with COVID-19, suffering from severe respiratory distress at increased risk of ICU admittance, who do not have any significant co-morbidities, which would place the subject at risk, or affect the ability to assess safety and efficacy, will be enrolled. Investigation of this patient population will provide relevant and valuable information on the treatment efficacies of the investigational agents.

22 Study activities

22.1 From day 1 until day 15

Study procedures	Screening Up to 72h prior to BL	Day 1 (BL)	Day 2	Day 3	Day 4	Day 5	Day 6	Day 7	Day 8	Day 9	Day 10	Day 11	Day 12	Day 13	Day 14	Day 15
Informed consent	x															
Eligibility criteria	x															
Patient information	x															
Vital signs assessment	x															
ECG assessment	x															
Physical examination	x															
Randomization		X														
SARS-CoV-test in NPH/sputum		X[a]														x
Weight/Height		X[b]														X[b]
Body Temperature[j]		X[d]	x	x	x	x	x	x	x	x	x	x	x	x	x	x
Hepatitis B and C test	X[c]															
HIV test	x															
BP, HR[j]		X[d]	x	x	x	x	x	x	x	x	x	x	x	x	x	X
ALT	X[e]	X[e]		X[e]		X[e]					X[e]					X[e]
LPK, Neutrophils (ANC) Lymphocyte count	X[e]	X[e]		X[e]		X[e]					X[e]					X[e]
Platelets	X[e]	X[e]		X[e]		X[e]					X[e]					X[e]
eGFR	X[e]	X[e]		X[e]		X[e]					X[e]					X[e]

Coagulation samples		X[e]		X[e]		X[e]					X[e]					X[e]
Respiratory frequency[j]		X[d]	x	x	x	x	x	x	x	x	x	x	x	x	x	x
CRP, Ferritin		X[e]		X[e]		X[e]					X[e]					X[e]
SARS-CoV-2 Virus levels		X		x		x					x					X
Routine clinical samples[j]	X[e]	X[e]		X[e]		X[e]					X[e]					X[e]
Blood test – culture for bacteria/fungi	x															
Arterial Po2		X[e]		X[e]		X[e]					X[e]					X[e]
SpO2/FiO2	x	x	x	x	x	x	x	x	x	x	x	x	x	x	x	x
SOFA score		X		x		x		x			x					X
NEWS2[j]		X[d]	x	x	x	x	x	x	x	x	x	x	x	x	x	X
Research samples / biobanking[j]		X		x		x					x					X
Prior and concomitant therapy assessment		X														
Adverse Event assessment[j]		X	x	x	x	x	X	x	x	x	x	x	x	x	x	X
Urine or serum pregnancy test	X[f]	X[f]														
8-point ordinal scale		X[g]														X
Lung x-ray	X[h]															
Pulmonary CT	x[i]															

a) SARS-CoV-2 test (positive/negative)
b) Height will be measured at Day 1 only. Weight should (if possible) be measured at the Day 1, 15, 29 and Early Termination (ET) occasion.
c) Performed at screening.
d) Performed day 1 and mandatory selected occasions (once daily).
e) Performed day 1 and mandatory selected occasions (same time in the morning), as well as additional timepoints, judged by clinical indication
f) Performed on all women of childbearing potential
g) Secondary clinical outcome: 8-point ordinal scale. Should be measured at the Day 1, 15 and 29 as well as at Early Termination (ET) occasion.
h) Not necessary if confirmed earlier, at the latest within 3 days
i) Should only be performed when clinical indication exists
j) Should only be performed in hospitalized patients, except for day 15 and 29 which are mandatory. Due to logistical issues, research samples and biobanking may be performed +/- 3 days from suggested sampling day.
k) Should be performed in all patients
l) End point for SAE assessments

Assessments can be performed at additional timepoints, judged by clinical indication.

22.2 From day 16 to day 60

Study procedures	Day 16	Day 17	Day 18	Day 19	Day 20	Day 21	Day 22	Day 23	Day 24	Day 25	Day 26	Day 27	Day 28	Day 29	Day 60/FU

SARS-CoV-test in NPH													X	
Weight/Height													X[b]	
Body Temperature[j]	X	X	x	x	x	x	x	x	x	x	x	x	x	X	
BP, HR[j]	X	X	x	x	x	x	x	x	x	x	x	x	x	x	
ALT[j]	X				X					X				X	
LPK, Neutrophils (ANC) Lymphocyte count[j]	X	X	x	x	x	x	x	x	x	x	x	x	x	x	
Platelets[j]	X	X	x	x	x	x	x	x	x	x	x	x	x	x	
eGFR[j]	X				X					X				X	
Respiratory frequency[j]	X	X	x	x	x	x	x	x	x	x	x	x	x	x	
CRP, Ferritin[j]	X				X					X				X	
SARS-CoV-2 Virus levels															
Routine clinical samples[j]	X				X					X				X	
Arterial Po2[j]															
SpO2/FiO2[j]	X	X	x	x	x	x	x	x	x	x	x	x	x	x	
SOFA score														x	
NEWS2[j]	X	X	x	x	x	x	x	x	x	x	x	x	x	x	
Research samples / biobanking														x	
Adverse Event assessment[j]	X	X	x	x	x	X	x	x	x	x	x	x	x	X[k]	X[l]
8-point ordinal scale														X[g]	

23 Study Procedures

23.1 Inclusion/Exclusion criteria

Subjects will be screened to ensure they meet all inclusion criteria and none of the exclusion criteria at both the screening and BL visits

23.2 Informed Consent

Signed informed consent will be obtained from the subject before any study procedures are undertaken.

23.3 Medical History

A complete medical and surgical history, as well as history of tobacco and alcohol use, will be obtained from each subject at the screening/inclusion visit.
All concomitant medication at the time of randomization will be recorded.
Additional medication that have been prescribed in the earlier 12 months and found of significance, as judged by the PI, will be recorded.

23.4 Blood pressure, heart rate, respiratory frequency, Body temperature

All measurements will be recorded in metric units when applicable.

23.5 Sequential organ failure assessment (SOFA)

The SOFA score is used to track a person's status during the stay in an intensive care unit (ICU) to determine the extent of a person's organ function or rate of failure. The score is based on six different scores (0-4), one each for the respiratory, cardiovascular, hepatic, coagulation, renal and neurological systems. The SOFA score rages from 0 to 24.

23.6 The National Early Warning Score 2 (NEWS2)

NEWS2 is used to improve the detection of and response to, clinical deterioration in patients with acute illness.

23.7 SARS-CoV-2 Test

NPH or sputum test. Performed at screening or < 3 days before screening.

23.8 SARS-CoV-2 levels

Performed day 1, at several occasions and at the endpoint 15 days and 29 days.

23.9 Pregnancy test

All female subjects will undergo a urine or serum pregnancy test, which will be performed at the screening. A lactating or pregnant female will not be eligible for participation in this trial.

23.10 Laboratory tests

The clinical laboratory tests (hematology, clinical chemistry and urinalysis) and serum pregnancy tests will be done at time points specified in table "Study activities until day 15" and "Study activities between day 16 and day 60" as well as on additional timepoints, if this is warranted due to clinical indication.

Blood samples for Hepatitis B and C as well as HIV serology will be obtained at Screening only. A subject will not be eligible for study participation if test results indicate positive acute or chronic Hepatitis B, C or HIV infection.

Additional blood samples will be obtained for research projects and biobanking. The blood samples for the research projects and biobanking will be obtained at screening, several study visits and at the endpoint.

23.11 Clinical Laboratory tests

Hematology:	WBC, lymphocytes, ANC (Differential count), Hemoglobin, Platelet count
Clinical Chemistry	IL-6, CRP, Ferritin, LD, PCT, ALT, Creatinine, Total bilirubin, AST, ALT. Myoglobin. Troponin T. CK-MB.

Coagulation samples	INR, APTT, D-dimer, Antithrombin, FDP, Fibrinogen, protein S, protein C (routine lab),<br>ROTEM (routine lab through the blood central/transfusion center)

Clinical assessments, regularly
Temperature / Fever
Oxygen supplementation, SpO2/FiO2
NEWS2 as recommended
Blood pressure, heart rate
Respiratory rate
Body mass (kg), Length (m), BMI
Use of high flow nasal cannula or mask ventilation(from which date, oxygen percentage, amount)

Assessments, endpoints
8-point ordinal scale (see above): at baseline and day 15
Number of days in mechanical ventilator / NIV / ECMO
Admitted to ICU (date, cause)
Death (date, cause of death)
Adverse events (date, specified AE)
Severe or life-threatening bacterial, invasive fungal, or opportunistic infection.

24 End of Study

The end of trial is defined as the last follow-up visit of the last recruited subject.

The study may be prematurely terminated due to a high number of serious adverse events related to the IMP or if the enrollment process cannot be completed within a reasonable time frame. Decision on premature study termination will be made by sponsor/coordinating investigator or because of a regulatory authority decision. If the study is prematurely terminated, the Investigator should promptly inform the patients and take necessary steps to finalize their engagement in the study. All relevant study material must be collected, and accountability completed.

Patients will be followed and treated by the treating physician according to standard of care as clinically indicated.

Within 90 days post study termination the form "Declaration of End of Trial Notification" should be submitted to the MPA and the Ethics committee.

25 Adverse events

The investigator will monitor each subject for clinical and laboratory evidence of adverse events (AEs) on a routine basis throughout the study. The investigator will assess and record any AE in detail including the date of onset, description, severity, duration and outcome, relationship of the AE to study drug, and any action(s) taken. For AEs to be considered sporadic, the events must be of

similar nature and severity. AEs observed by site personnel, or reported spontaneously by the subject will be recorded. All AEs will be followed to a satisfactory conclusion.

An AE is defined as any untoward medical occurrence in a subject or clinical investigation subject administered a pharmaceutical product and which does not necessarily have a causal relationship with this treatment. An AE can therefore be any unfavorable and unintended sign (including an abnormal laboratory finding), symptom, or disease temporally associated with the use of a medicinal (investigational) product, whether or not the event is considered causally related to the use of the product.
Such an event can result from use of the drug as stipulated in the protocol or labeling, as well as from accidental or intentional overdose or drug withdrawal. Any worsening of a pre-existing condition is considered an AE. Laboratory abnormalities and changes in vital signs are considered to be AEs only if they result in discontinuation from the study, necessitate therapeutic medical intervention, and/or if the investigator considers them to be AEs. A treatment-emergent AE is defined as any AE with onset or worsening reported by a subject from the time that the first dose of study drug is administered.

25.1 Serious Adverse event

Each AE is to be classified by the investigator as serious or non-serious. Seriousness is not defined by a medical term; it is a result or an outcome. An AE is defined as a Serious Adverse Event (SAE) if it:

- results in death
- is life-threatening
- requires inpatient hospitalisation or prolongation of existing hospitalisation
- results in persistent or significant disability/incapacity
- results in a congenital anomaly/birth defect
- other medically important event

If an AE meets any of the above criteria, it is to be reported to Sponsor as a serious adverse event (SAE) immediately or within 24 hours of the site being made aware of the SAE.

25.2 Adverse Event Severity

The investigator will use the following definitions to rate the severity of each AE:

Mild The AE is transient and easily tolerated by the subject.

Moderate The AE causes the subject discomfort and interrupts the subject's usual activities.

Severe The AE causes considerable interference with the subject's usual activities and may be incapacitating or life-threatening.

25.3 Relationship to Study Drug

The investigator will use the following definitions to assess the relationship of the AE to the use of study drug:

Probably Related An AE has a strong temporal relationship to study drug or recurs on re-challenge and another etiology is unlikely or significantly less likely.

Probably Not Related An AE has little or no temporal relationship to the study drug and/or a more likely alternative etiology exists.

Not Related An AE is due to an underlying or concurrent illness or effect of another drug and is not related to the study drug (*e.g.*, has no temporal relationship to study drug or has a much more likely alternative etiology).
If an investigator's opinion of possibly, probably not, or not related to study drug is given, an alternate etiology must be provided by the investigator for the AE.

25.4 Adverse Event Collection Period

All AEs reported from the time of study drug administration until day 60 of the study will be collected, whether elicited or spontaneously reported by the subject. In addition, SAEs will be collected from the time the subject signed the study-specific informed consent. Information on SAEs and AEs is collected at each clinic visit.

25.5 Serious Adverse Drug Reaction Reporting

A serious adverse drug reaction (SADR) can be a suspected unexpected serious adverse reaction (SUSAR), which means that may or may not be dose related, but are unexpected, as they are not consistent with current information. If the sponsor judge that a SAE is SUSAR the sponsor is responsible for reporting it to the medical products agency and the local ethics committee. As the Sponsor is not able to report electronically to the EudraVigilance database, SUSAR will be reported using the CIOMS form (https://cioms.ch/wp-content/uploads/2017/05/cioms-form1.pdf) which will be sent to the Medical Products Agency. The Medical Products Agency will then do the reporting into the EudraVigilance database. A SUSAR that is life-threatening must be reported to the medical products agency promptly or within 7 days after occurrence or sponsor awareness (15 days in case of not life-threatening SUSAR).

26 Risk Benefit justification

As with all drugs there are side effects from use of Kineret and RoActemra, of which some can be severe and even life threatening. A complete list can be found in the SmPC for each product respectively. Also, emergent side effects in a new patient population cannot be ruled out. However, serious side effects of the two drugs beside infections are rare and current literature concerning ongoing controlled studies and off-label use in COVID-19 has not suggested any serious side-effects to our knowledge. Lack of any obvious side effects is also confirmed from local experience of off-label use of Tocilizumab in 35 patients and of Anakinra in a handful of patients at Karolinska. Given the life-threatening nature of a severe COVID-19 infection, the current lack of efficient treatments, and the potential benefits from immunomodulating drugs, the risks are therefore considered acceptable. Furthermore, patients are clinically evaluated daily and blood sampled at short intervals during the study period in order to catch possible side effects and avoid exacerbation due to continued administration of study drugs. As a precaution to avoid severe bacterial infections due

to immune suppression, all patients will be treated with broad spectrum antibiotics. For the purposes of regulatory reporting, all serious adverse reactions will be subject to expedited reporting. The specification of and frequency of laboratory tests is justified by the clinical condition as well as monitoring of study treatments.

27 Protocol Deviations

When a deviation from the protocol is deemed necessary for an individual subject, the investigator or other physician in attendance must discuss this with the sponsor to decide as to whether or not the subject is allowed to enter and/or continue the study.
The approval authorized will only apply for that subject. A note-to-file should be written and filed in the Study Master File. If the sponsor deems necessary, a change in the protocol may be implemented. This has to be approved by the IEC/IRB and regulatory agencies, as applicable.

28 Coding and tabulation of Adverse Events

All adverse events (AEs) will be coded and tabulated by body system preferred term for individual events within each body system and will be presented in descending frequency. AEs will also be tabulated by severity and relationship to the study medication. Serious adverse events (SAEs) will be summarized separately. AEs and laboratory abnormalities encountered during the study will be followed until resolution or stabilization.

29 Independent Ethics Committee (IEC)

Good Clinical Practice (GCP) requires that the clinical protocol, any protocol amendments, the Investigator's Brochure, the informed consent and all other forms of subject information related to the study and any other necessary documents be reviewed by an IEC. IEC approval of the protocol, informed consent and subject information as relevant, will be obtained prior to study start.

Any amendments to the protocol will require IEC approval prior to implementation of any changes made to the study design.

During the conduct of the study, the investigator should promptly provide written reports (*e.g.,* International Conference on Harmonization (ICH) Expedited Reports, and any additional reports required by local regulations) to the IEC of any changes that affect the conduct of the study and/or increase the risk to subjects.

30 Ethical Conduct of the Study

The study will be conducted in accordance with the protocol, ICH guidelines, applicable regulations and guidelines governing clinical study conduct and the ethical principles that have their origin in the Declaration of Helsinki.

31 Subject Information and Consent

The investigator or his/her representative will explain the nature of the study to the subject, and answer all questions regarding this study. Prior to any study-related screening procedures being performed on the subject, the informed consent statement will be reviewed and signed and dated by the subject and the person who administered the informed consent. A copy of the informed consent form will be given to the subject and the original will be placed in the subject's medical record. An entry must also be made in the subject's dated source documents to confirm that informed consent was obtained prior to any study-related procedures and that the subject received a signed copy.

32 Source Documents

Source documents are defined as original documents, data and records. This may include hospital records, clinical and office charts, laboratory data/information, subjects' diaries or evaluation checklists, pharmacy dispensing and other records, recorded data from automated instruments, microfiches, photographic negatives, microfilm or magnetic media, and/or x-rays. Data collected during this study must be recorded on the appropriate source documents, as specified in the study specific source data log.

33 Case Report Forms

The study will use electronic CRFs. The principal investigator will review the CRFs for completeness and accuracy and sign and date each set of CRFs where indicated. The CRFs will be reviewed periodically for completeness by study personnel. Study personnel will be allowed access to all source documents in order to verify CRF entries.

34 Monitoring

In order to assure that the study is conducted in accordance with the study protocol, that study data is collected properly, that documentation and reporting is done in accordance with ICH-GCP (Good Clinical Practice) and additional ethical and regulatory requirements, the study will be monitored by a qualified external professional prior to study start, during, and after study termination. An additional aim of the monitoring is to reassure the safety and integrity of the study subjects, and to ensure that study data is correctly and completely collected in the CRF and correspond to source data. The investigator and the head of the medical institution (where applicable) agrees to allow the monitor direct access to all relevant documents.

To ensure compliance with GCP and all applicable regulatory requirements, regulatory agencies may conduct a regulatory inspection of this trial. Such audits/inspections can occur at any time during or after completion of the trial. If an audit or inspection occurs, the investigator and institution agree to allow the auditor/inspector direct access to all relevant documents and to allocate his/her time and the time of his/her staff to the auditor/inspector to discuss findings and any relevant issues.

35 Archiving

All data will be registered, managed, and stored in a manner that enables correct reporting, interpretation, and verification. The complete Trial Master File, as well as source documents, will be archived for at least 10 years after the study is completed. Source data in the medical records system is stored and archived in accordance with the respective hospital regulations.

36 References

1. Zhou F et al, Lancet 2020

2. WHO. Novel coronavirus (COVID-19) situation. Updated March 24, 2020. https://experience.arcgis.com/experience/685d0ace521648f8a5beeeee1b9125cd

3. Wu Z, McGoogan JM. Characteristics of and important lessons from the coronavirus disease 2019 (COVID-19) outbreak in China: summary of a report of 72 314 cases from the Chinese Center for Disease Control and Prevention. *JAMA*. 2020. Published online February 24, 2020. doi:10.1001/jama.2020.2648

4. Lu H. Drug treatment options for the 2019-new coronavirus (2019-nCoV). *Biosci Trends*. 2020;14(1):69-71.

5. Huang C, Wang Y, Li X, et al. Clinical features of patients infected with 2019 novel coronavirus in Wuhan, China. *Lancet*. 2020;395(10223):497-506.
6. Zhang C et al, Int J of Antimicrobial Agents, 2020
7. Concetta Castilletti, Licia Bordi, Eleonora Lalle, *et al.*Coordinate induction of IFN-alpha and -gamma by SARS-CoV also in the absence of virus replication
Virology, 341 (1) (2005), pp. 163-169

8. Shu-Qun Shi, Jing-Pian Peng, Yin-Chuan Li, *et al.*The expression of membrane protein augments the specific responses induced by SARS-CoV nucleocapsid DNA immunization
Molecular immunology, 43 (11) (2006), pp. 1791-1798

9. Chien-Te K. Tseng, Lucy A. Perrone, Hongbing Zhu, *et al.*Severe acute respiratory syndrome and the innate immune responses: modulation of effector cell function without productive infection
Journal of immunology, 174 (12) (2005), pp. 7977-7985

10. Fung SY, et al. A tug-of-war between severe acute respiratory syndrome coronavirus 2 and host antiviral defence: lessons from other pathogenic viruses. Cell Biosci. 2020 Mar 16;10:40.

11. Y. Zheng, Q.Y. WangBioinformatics analysis on molecular mechanism of ribavirin and interferon-α in treating MERS-CoV
Zhonghua liuxingbingxue zazhi, 37 (2) (2016), pp. 291-293

12. Emile JF et al, Revised classification of histiocytoses and neoplasms of the macrophage-dendritic cell lineages. Blood. 2016 Jun 2;127(22):2672-81.

13. La Rosée P, Horne AC, Hines M, von Bahr Greenwood T, Machowicz R, Berliner N, Birndt S, Gil-Herrera J, Girschikofsky M, Jordan MB, Kumar A, van Laar JAM, Lachmann G, Nichols KE, Ramanan AV, Wang Y, Wang Z, Janka G, Henter JI. Blood. 2019 Jun 6;133(23):2465-2477.

14. Sönmez HE, Demir S, Bilginer Y, Özen S. Clin Rheumatol. 2018 Dec;37(12):3329-3335. doi: 10.1007/s10067-018-4095-1. Epub 2018 Apr 16. Review.
15. Boomer JS, To K, Chang KC, et al. Immunosuppression in patients who die of sepsis and multiple organ failure. JAMA 2011;306:2594-605.

16. Patil NK, Bohannon JK, Sherwood ER. Imunotherapy: A promising approach to reverse sepsis-induced immunosuppression. Pharmacol Res 2016; 111; 688-702

17. Hotchkiss RS, Monneret G, Payen D. Sepsis-induced immunosuppression: from cellular dysfunctions to immunotherapy. Nature reviews Immunology 2013;13:862-74.

18. Gille Johnsson. Diagnostic and prognostic markers in sepsis. Thesis Karolinska Institutet 2013

19. Shakoory B, Carcillo JA, Chatham WW, Amdur RL, Zhao H, Dinarello CA, Cron RQ, Opal SM Crit Care Med 2016: Feb;44(2):275-81.

20 Scheller J, Rose-John S: Medical microbiology and immunology, 195 (4) (2006), pp. 173-183

21. Geraldine Navarro, Sara Taroumian, Nashla Barroso, *et al.*Tocilizumab in rheumatoid arthritis: a meta-analysis of efficacy and selected clinical conundrums Seminars in arthritis and rheumatism, 43 (4) (2014), pp. 458-469

22. Shumpei Yokota, Takako Miyamae, Tomoyuki Imagawa, *et al.*Therapeutic efficacy of humanized recombinant anti-interleukin-6 receptor antibody in children with systemic-onset juvenile idiopathic arthritis
Arthritis and rheumatism, 52 (3) (2005), pp. 818-825

23. Campbell L, Chen C, Bhagat SS, Parker RA, Ostor AJ. Risk of adverse events including serious infections in rheumatoid arthritis patients treated with tocilizumab: a systematic literature review and meta-analysis of randomized controlled trials. Rheumatology (Oxford) 2011; 50; 552-562

24. Geng Z, Yu Y, Hu S, Dong L, Ye C, Tocilizumab and the risk of respiratory adverse events in patients with rheumatoid arthritis: a systematic review and meta-analysis of randomised controlled trials. Clinical and experimental rheumatology 2019; 37:;318-323

25. Stephan A. Grupp, Michael Kalos, David Barrett, *et al.*Chimeric antigen receptor-modified T cells for acute lymphoid leukemia
The New England journal of medicine, 368 (16) (2013), pp. 1509-1518

26. Xu X, et al. Effective treatment of severe COVID-19 patients with tocilizumab. ChinaXiv: 202003.00026v1

27. SISCO Study (NCT04322188); https://www.clinicaltrials.gov/ct2/show/NCT04322188

28. Lai TL, Lavori PW, Shih MC. Adaptive trial designs. Annu Rev Pharmacol Toxicol. 2012;52:101–110. doi:10.1146/annurev-pharmtox-010611-134504

29. Pallmann, P., Bedding, A.W., Choodari-Oskooei, B. *et al.* Adaptive designs in clinical trials: why use them, and how to run and report them. *BMC Med* 16, 29 (2018). https://doi.org/10.1186/s12916-018-1017-7

30. Bhatt DL et al, N Engl J Med 2016; 375:65-74

31. Opal SM, Fisher CJ Jr, Dhainaut JF, et al. Crit Care Med. 1997;25:1115–1124
