# Supplementary material for: Anakinra or tocilizumab in patients admitted to hospital with severe covid-19 at high risk of deterioration (IMMCoVA): A randomized, controlled, open-label trial
Source: PLoS One. 2023 Dec 29;18(12):e0295838. doi: 10.1371/journal.pone.0295838 (PMC10756513; doi:10.1371/journal.pone.0295838)
Supplement: S2 File — (DOCX) [file pone.0295838.s006.docx]

**S2 File. Protocol amendments during study**

The protocol was ethically approved (2020-01973) and then amended (2020-02530) before inclusion of first patients according to protocol version 4.0, online supplement. After inclusion of two patients (one anakinra, one usual care), the protocol was amended, Study Protocol ImmCoVA v5.0, in July 2020 (2020-04082, approved in September) with the following changes:

- The study was expanded from single to multiple centers.
- Steroid treatment was allowed in all arms since it had been introduced as part of usual care.
- Antibiotic prophylaxis was only prescribed in the anakinra and tocilizumab arms, not in the usual care arm.
- Inclusion criteria were adjusted – differences compared to original protocol:
  - Age limit was extended to all patients aged 18 and over (previously, 18-80).
  - Respiratory criterion: Previously “PaO2/FiO2 < 26,8 kPa (200 mm Hg) for at at least 8 hours, corresponding to 5 liters/minute of Oxygen to maintain SpO2 at 94%. The patient must also have an increased breathing frequency ≥ 25 / min.” was changed to: “5 liters/minute of Oxygen for at least 8 hours to maintain SpO2 at ≥93%. A shorter duration is also accepted if presentation is acute, and the patient needs more than 10 liters/minute of Oxygen to maintain SpO2 at ≥93%.”
  - Time limits for ferritin and CRP elevation as inclusion criteria were clarified
  - Time limit for individual measures on zero to three-point inclusion scale were specified
- Exclusion criteria were adjusted: Acceptable period of steroid treatment before inclusion was specified to five days.
- Secondary endpoints were clarified: Time points for measurement of various endpoints were clarified
- Secondary endpoints were added: fraction of patients discharged to institution other than normal domicile; number of patients admitted to ECMO, ICU and HDU; total steroid use during study.

A final amendment was approved in November 2020 (2020-05709), concerning expansion of study sites to include Uppsala.
