# Supplementary material for: Anakinra or tocilizumab in patients admitted to hospital with severe covid-19 at high risk of deterioration (IMMCoVA): A randomized, controlled, open-label trial
Source: PLoS One. 2023 Dec 29;18(12):e0295838. doi: 10.1371/journal.pone.0295838 (PMC10756513; doi:10.1371/journal.pone.0295838)
Supplement: S3 File — (DOCX) [file pone.0295838.s007.docx]

**S3 File. Recruitment interruption and adherence to protocol**

**Interruption of recruitment**

The study was temporarily paused pending review by the data and safety monitoring board between Dec 6-17 2020.

**Adherence to protocol**

All patients received assigned therapy, except two patients allotted to Anakinra who both received study drug for four instead of seven days, one whom declined further drug administration but agreed to otherwise participate in follow-up. The other patient had rising AST and ALT wherefore anakinra was discontinued. Liver involvement was ultimately deemed to be caused by covid-19 (enzymes were already elevated at inclusion). Both patients were included in the anakinra group in analyses.
