# Supplementary material for: Anakinra or tocilizumab in patients admitted to hospital with severe covid-19 at high risk of deterioration (IMMCoVA): A randomized, controlled, open-label trial
Source: PLoS One. 2023 Dec 29;18(12):e0295838. doi: 10.1371/journal.pone.0295838 (PMC10756513; doi:10.1371/journal.pone.0295838)
Supplement: S4 File — (DOCX) [file pone.0295838.s008.docx]

**S4 File. Causes of death**

There were 6 deaths up to day 29, resulting in a total mortality rate up to day 29 of 7%. Two patients died in the usual care group (staphylococcus septicaemia, aspiration pneumonitis), 2 patients in anakinra group (Severe covid progression and multiorgan failure and in one patient sudden death after Covid recovery*) and 2 patients in the tocilizumab group (both with severe progress in Covid with severe resp failure). Two additional patients died on or after d29 (one in the usual care group with severe covid progression and multiorgan failure, d29, and one in the anakinra group with severe covid progression and multiorgan failure, d43). No deaths were considered by the investigators to be related to treatment assignment. (p=1.0 for UC vs anakinra and 0.9 for UC vs tocilizumab).
