# Supplementary material for: Anakinra or tocilizumab in patients admitted to hospital with severe covid-19 at high risk of deterioration (IMMCoVA): A randomized, controlled, open-label trial
Source: PLoS One. 2023 Dec 29;18(12):e0295838. doi: 10.1371/journal.pone.0295838 (PMC10756513; doi:10.1371/journal.pone.0295838)
Supplement: Attachment — Submitted filename: Response to reviewers .docx [file pone.0295838.s010.docx]

Dear editor,

Please find our point-by-point responses to requests made by PLOS ONE and to questions and suggestions by the reviewer. Our responses are in **bold layout.**

At this time, please address the following queries:

a)        Please clarify the sources of funding (financial or material support) for your study. List the grants or organizations that supported your study, including funding received from your institution. **The funder (Swedish Research Council) has been listed.**

b)        State what role the funders took in the study. If the funders had no role in your study, please state: “The funders had no role in study design, data collection and analysis, decision to publish, or preparation of the manuscript.” **The text has been changed accordingly, line 448-449.**

c)        If any authors received a salary from any of your funders, please state which authors and which funders. **No salary was paid by funders.**

d)        If you did not receive any funding for this study, please state: “The authors received no specific funding for this work.” **Not applicable**

Please include your amended statements within your cover letter; we will change the online submission form on your behalf. **We have specified changes in the cover letter.**

3. We note that the grant information you provided in the ‘Funding Information’ and ‘Financial Disclosure’ sections do not match. **We have now made sure the sections match. The text now reads: “This academic study was funded by a grant from the Swedish Research Council (2020-06318 JL;JSC;AS). JSC also received funding from Center for Innovative Medicine.” Line 469-470. However, we haven´t figured out how to change to the same text in the financial disclosure section.**

When you resubmit, please ensure that you provide the correct grant numbers for the awards you received for your study in the ‘Funding Information’ section. **The grant number has been added to the manuscript. Line 447**

4. In your Data Availability statement, you have not specified where the minimal data set underlying the results described in your manuscript can be found. PLOS defines a study's minimal data set as the underlying data used to reach the conclusions drawn in the manuscript and any additional data required to replicate the reported study findings in their entirety. All PLOS journals require that the minimal data set be made fully available. For more information about our data policy, please see <http://journals.plos.org/plosone/s/data-availability>. **The data set has now been added as Study data - Supporting Information files.**

5. Your ethics statement should only appear in the Methods section of your manuscript. If your ethics statement is written in any section besides the Methods, please move it to the Methods section and delete it from any other section. Please ensure that your ethics statement is included in your manuscript, as the ethics statement entered into the online submission form will not be published alongside your manuscript. **The ethics statement has been moved to the method section.**

6. Please review your reference list to ensure that it is complete and correct. If you have cited papers that have been retracted, please include the rationale for doing so in the manuscript text, or remove these references and replace them with relevant current references. Any changes to the reference list should be mentioned in the rebuttal letter that accompanies your revised manuscript. If you need to cite a retracted article, indicate the article’s retracted status in the References list and also include a citation and full reference for the retraction notice. **We have reviewed the reference list and believe that it is complete and correct. We found no retracted papers in the list.**

**Reviewers' comments:**

Reviewer's Responses to Questions

**First, thank you for a careful review and valuable comments, questions and suggestions which have resulted in changes that we believe has improved the manuscript!**

**Comments to the Author**

1. Is the manuscript technically sound, and do the data support the conclusions?

The manuscript must describe a technically sound piece of scientific research with data that supports the conclusions. Experiments must have been conducted rigorously, with appropriate controls, replication, and sample sizes. The conclusions must be drawn appropriately based on the data presented.

Reviewer #1: Yes

2. Has the statistical analysis been performed appropriately and rigorously?

Reviewer #1: Yes

3. Have the authors made all data underlying the findings in their manuscript fully available?

The [PLOS Data policy](http://www.plosone.org/static/policies.action#sharing) requires authors to make all data underlying the findings described in their manuscript fully available without restriction, with rare exception (please refer to the Data Availability Statement in the manuscript PDF file). The data should be provided as part of the manuscript or its supporting information, or deposited to a public repository. For example, in addition to summary statistics, the data points behind means, medians and variance measures should be available. If there are restrictions on publicly sharing data—e.g. participant privacy or use of data from a third party—those must be specified.

Reviewer #1: No

4. Is the manuscript presented in an intelligible fashion and written in standard English?

PLOS ONE does not copyedit accepted manuscripts, so the language in submitted articles must be clear, correct, and unambiguous. Any typographical or grammatical errors should be corrected at revision, so please note any specific errors here.

Reviewer #1: Yes

5. Review Comments to the Author

Please use the space provided to explain your answers to the questions above. You may also include additional comments for the author, including concerns about dual publication, research ethics, or publication ethics. (Please upload your review as an attachment if it exceeds 20,000 characters)

Reviewer #1: This is an interesting RCT assessing the clinical efficacy and safety of tocilizumab and anakinra among hospitalized adults with laboratory-confirmed covid-19 at high risk of deterioration.
**Thank you!**
Some main points to consider:

Randomisation was stratified by study site, sex and age. Understandably the recruitment was discontinued leading to an impact in imbalance, however they seems to be imbalance between groups for sex. Was there adjustment done for this in the analysis? **No, all analyses were unadjusted.** **We have added a sensitivity analysis adjusted for sex and age for the primary outcome, lines 224-226 and 312-316.**

The study shows that in addition an adaptive design approach was implemented for the re-adjustment of sample size at 50% of subjects recruited and an interim analysis performed. Suggest the authors also include the pre-specified alpha spent at this look at the data. This should be mentioned in the methods. **Since this was a blinded analysis for the sole purpose of recalculating sample size, we considered it to have limited effect on the type I error, and that the interim analysis thus spent no alpha.** .

Other minor point to consider for clarification of the manuscript.

1. In the sample size calculation statement (line 199) include the SD (assumed to be 6 when recalculating the sample size). Thank you for pointing this out. The text **has been amended and now reads “Taking the significance level at 5% and Type II error as 20% and an expected mean difference of 4 days, while assuming a standard deviation of 6, 36 subjects were required per group to obtain 80% power. “ Line 201.**
2. In the statistical analysis section - define your population of analysis i.e ITT since this is stated in the results section. i.e analysed as they were randomised etc..**The text has been amended and now reads “Analyses were performed by intention-to-treat, i e analysed as randomized.” Line 216**
3. Define your safety population - e.g those who took at least one dose of treatment or started treatment etc. **The text has been amended and now reads “The safety population was defined according to initiation of either study drug or usual care only” Line 183-184**
4. Suggest to rename table 1 to "Table 1 Patient characteristics at baseline by randomised groups" **Thank you for the suggestion. The table has been renamed. Line 270.**
5. This is an open label study, mention finalization of the statistical analysis plan i.e prior to first patient recruitment was this done? **The following has been specified: “The statistical analysis plan was finalized prior to recruitment of the first patient.” Line 192.**
6. Mention handling of missing data in the stats section. **This information has been added: “Missing data were handled as missing at random.” Line 218**

**Sincerely,**

**Jonas Sunden-Cullberg, PI**
